# Effect of artificial intelligence-aided differentiation of adenomatous and non-adenomatous colorectal polyps at CT colonography on radiologists’ therapy management

**DOI:** 10.1007/s00330-025-11371-0

**Published:** 2025-01-25

**Authors:** Sergio Grosu, Matthias P. Fabritius, Michael Winkelmann, Daniel Puhr-Westerheide, Maria Ingenerf, Stefan Maurus, Anno Graser, Christian Schulz, Thomas Knösel, Clemens C. Cyran, Jens Ricke, Philipp M. Kazmierczak, Michael Ingrisch, Philipp Wesp

**Affiliations:** 1 Department of Radiology, LMU University Hospital, LMU Munich, Marchioninistraße 15, 81377 Munich, Germany; 2 Department for Diagnostic and Interventional Radiology and Neuroradiology, Klinikum Kempten, Robert-Weixler-Straße 50, 87439 Kempten, Germany; 3 Gemeinschaftspraxis Radiologie München, Burgstraße 7, 80331 Munich, Germany; 4 Department of Medicine II, LMU University Hospital, LMU Munich, Marchioninistraße 15, 81377 Munich, Germany; 5 Department of Pathology, LMU University Hospital, LMU Munich, Marchioninistraße 15, 81377 Munich, Germany; 6 Munich Center for Machine Learning (MCML), Geschwister-Scholl-Platz 1, 80539 Munich, Germany

**Keywords:** CT Colonography, Polyps, Machine learning, Cancer screening

## Abstract

**Objectives:**

Adenomatous colorectal polyps require endoscopic resection, as opposed to non-adenomatous hyperplastic colorectal polyps. This study aims to evaluate the effect of artificial intelligence (AI)-assisted differentiation of adenomatous and non-adenomatous colorectal polyps at CT colonography on radiologists’ therapy management.

**Materials and methods:**

Five board-certified radiologists evaluated CT colonography images with colorectal polyps of all sizes and morphologies retrospectively and decided whether the depicted polyps required endoscopic resection. After a primary unassisted reading based on current guidelines, a second reading with access to the classification of a radiomics-based random-forest AI-model labelling each polyp as “non-adenomatous” or “adenomatous” was performed. Performance was evaluated using polyp histopathology as the reference standard.

**Results:**

77 polyps in 59 patients comprising 118 polyp image series (47% supine position, 53% prone position) were evaluated unassisted and AI-assisted by five independent board-certified radiologists, resulting in a total of 1180 readings (subsequent polypectomy: yes or no). AI-assisted readings had higher accuracy (76% +/− 1% vs. 84% +/− 1%), sensitivity (78% +/− 6% vs. 85% +/− 1%), and specificity (73% +/− 8% vs. 82% +/− 2%) in selecting polyps eligible for polypectomy (*p* < 0.001). Inter-reader agreement was improved in the AI-assisted readings (Fleiss’ kappa 0.69 vs. 0.92).

**Conclusion:**

AI-based characterisation of colorectal polyps at CT colonography as a second reader might enable a more precise selection of polyps eligible for subsequent endoscopic resection. However, further studies are needed to confirm this finding and histopathologic polyp evaluation is still mandatory.

**Key Points:**

***Question*** *This is the first study evaluating the impact of AI-based polyp classification in CT colonography on radiologists’ therapy management.*

***Findings*** *Compared with unassisted reading, AI-assisted reading had higher accuracy, sensitivity, and specificity in selecting polyps eligible for polypectomy.*

***Clinical relevance*** *Integrating an AI tool for colorectal polyp classification in CT colonography could further improve radiologists’ therapy recommendations.*

**Graphical Abstract:**

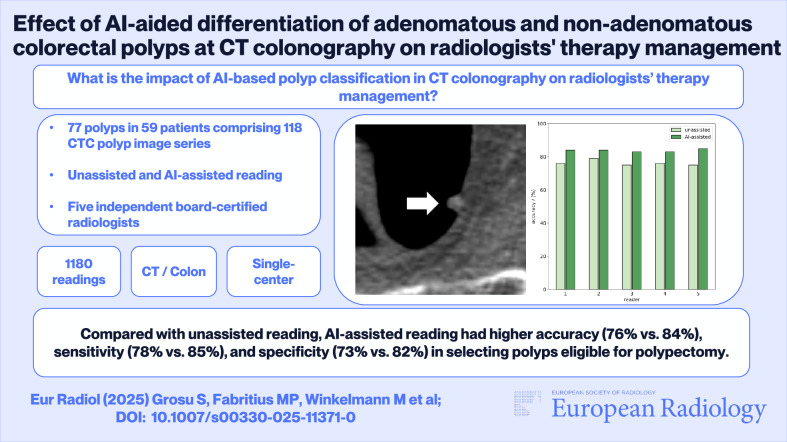

## Introduction

Colorectal cancer is one of the three leading cancer-related causes of death in industrialised countries [1]. The incidence and mortality of colorectal cancer can be significantly reduced by early detection and endoscopic resection of adenomatous polyps of the colon since most types of colorectal cancer develop from these over several years [2–5]. Therefore, screening procedures, especially optical colonoscopy, play an essential role in the prevention of colorectal cancer [6].

Computed tomography colonography is a non-invasive screening method for the early detection of colorectal cancer, whose sensitivity is comparable to optical colonoscopy in detecting colorectal polyps larger than 5 mm [7–9]. CT colonography has the benefit of visualising segments of the colon beyond strictures or complex anatomy, no sedation is required, and participation rates are higher compared to optical colonoscopy [10–13].

CT colonography, however, does not allow a clear differentiation between non-adenomatous and adenomatous colorectal polyps. This distinction would be essential for therapy management since adenomatous polyps require endoscopic resection, in contrast to non-adenomatous hyperplastic polyps, where unnecessary procedures should be avoided. European guidelines on CT colonography (European Society of Gastrointestinal Endoscopy, European Society of Gastrointestinal and Abdominal Radiology) recommend endoscopic resection of colorectal polyps ≥ 6 mm with the option for surveillance only where optical colonoscopy is not feasible [14]. The CT Colonography Reporting and Data System (C-RADS) of the American College of Radiology following United States guidelines (United States Multi-Society Task Force on Colorectal Cancer, American Cancer Society) recommends endoscopic resection of colorectal polyps ≥ 10 mm. To patients with colorectal polyps of 6–9 mm who can undergo optical colonoscopy, polypectomy may be offered, and in other patients with one or two polyps of 6–9 mm, follow-up at 3 years may be considered depending on age and comorbidity [14–17]. First studies have shown that an artificial intelligence (AI)-based evaluation of CT colonography images may allow a non-invasive distinction between non-adenomatous and adenomatous colorectal polyps [18–20]. For instance, a radiomics-based random-forest machine-learning model, which was also used in the present study, enabled the robust differentiation of non-adenomatous and adenomatous colorectal polyps in CT colonography with an AUC of 0.91, interestingly with only one feature assessing polyp size among the 10 most important image features for decision-making, ranked in fourth place [18]. However, these studies did not evaluate the radiologist-AI interaction in a clinical scenario.

It was shown that computer-aided detection algorithms used as a second reader could reduce the number of missed colorectal polyps during CT colonography [21–24]. However, these studies evaluated polyp detection only, not polyp classification. There is a lack of data on the impact of computer-aided classification of CT colonography-detected colorectal polyps on therapy management. Even a well-functioning model is useless if it does not offer added value for radiologists’ clinical decision-making.

The aim of this study was to evaluate the effect of AI-aided differentiation of non-adenomatous and adenomatous colorectal polyps at CT colonography on radiologists’ therapy management using a radiomics-based random-forest machine-learning model analysing polyp characteristics beyond size and morphology [18].

## Materials and methods

### Study population

This retrospective study was approved by the institutional review board, and the requirement for written informed consent was waived. The CT colonography datasets from a North American multicentre CT colonography screening trial are publicly available via The Cancer Imaging Archive (TCIA) [25–27]. The Cancer Imaging Archive is a multicentre, open-source, open-access collection of anonymised medical images of cancer. The CT colonography images were acquired with varying scanning protocols on multiple CT scanners from several vendors (Canon Medical Systems, GE Healthcare Systems, Philips Healthcare, Siemens Healthineers). Only polyps with available histopathologic reports were included [18]. A sample size calculation for McNemar’s two-sample paired-proportions test revealed a required sample size of 85 comparisons (polyp image series) for 5 readers under the assumptions of a success-failure proportion for polypectomy recommendation of 5% and a failure-success proportion of 25% (power = 80%, one-sided alpha = 0.01) [28]. The dataset was used for external validation in a previously published study investigating the AI-based differentiation of non-adenomatous and adenomatous colorectal polyps detected with CT colonography [18]. This study differs from the previous work, as it evaluates the effect of AI-aided polyp differentiation as a second reader for radiologists, and not AI-model performance.

### CT colonography dataset preparation

**Fig. 1:**
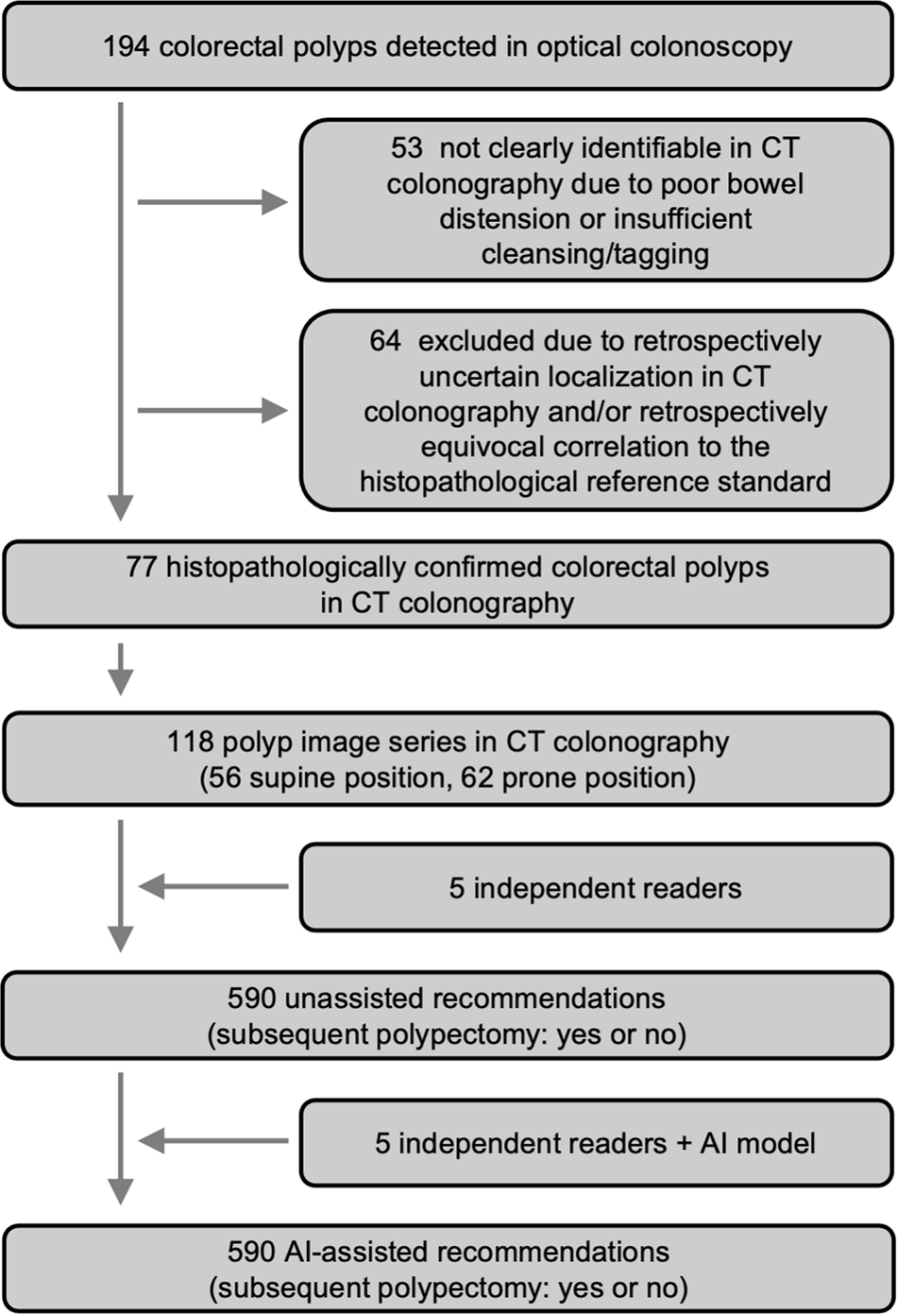
Flow diagram of the study population

In the CT colonography datasets (Fig. 1) available via TCIA, prospective colorectal polyp detection was performed by 15 radiologists who had participated in specialised training on CT colonography, had read > 500 CT colonography examinations prior to the study, and had passed a qualifying examination [25–27]. A polyp was rated as a true positive detection if it was localised in the same colonic segment in CT colonography and optical colonoscopy, and if the measured size of the polyp was within 50% of its reference standard measure derived from histopathology or optical colonoscopy in case of piecemeal resection, as described in detail before [25–27].

The information obtained on polyp size and localisation was used for retrospective polyp re-detection and segmentation performed by a board-certified radiologist (500–750 CT colonography cases’ experience; having completed a specialised hands-on workshop on CT colonography) and two radiology residents (50–500 CT colonography cases experience; one having completed a specialised hands-on workshop on CT colonography) blinded to histopathological polyp class [18]. The dedicated post-processing software syngo.via versionVA30B (Siemens Healthineers) was used for polyp detection. The Medical Imaging Interaction Toolkit (MITK) Version 2018.04 (German Cancer Research Centre, Division of Medical Image Computing) was used for polyp segmentation [29]. In the case of divergent reading results, consensus reading was performed. Consensus was reached when all readers agreed on polyp localisation and segmentation. Colorectal polyps that could not be clearly identified in CT colonography and/or unequivocally assigned to the corresponding histopathological report were excluded. Each polyp was segmented on images in supine and prone positions if confidently detectable in both positions. Polyps of all size categories and morphologies were included, as described in detail before [18].

### Artificial intelligence algorithm

The CT colonography scans and segmentation masks were resampled to a pixel spacing of 0.72 mm and slice thickness of 0.5 mm. For each scan, 1906 radiomic image features describing grey-level histogram statistics, texture, and shape were extracted from the segmented voxels using the open-source Python package Pyradiomics (version 2.2.0; Harvard Medical School). Feature correlation was quantified by calculating a Pearson correlation matrix for all features and only the 198/1906 (10%) least correlated features were used for analysis.

A random-forest machine-learning model classified each polyp segmentation in the dataset as non-adenomatous or adenomatous based on the 198 radiomic features. The machine-learning model was previously trained on a separate CT colonography training dataset comprising 169 manual polyp segmentation and binary ground truth labels (non-adenomatous or adenomatous) for each polyp based on histopathological reports. Model training and the training dataset were described in detail before [18]. The trained model, as well as the code for training and application, are publicly available on the platform GitHub (https://github.com/pwesp/random-forest-polyp-classification).

### Colorectal polyp analysis

Image analysis was performed by 5 independent board-certified radiologists. One reader had 500–750 CT colonography cases experience, 4 readers had 50–500 CT colonography cases experience. One reader completed a specialised hands-on workshop on CT colonography. The CT colonography workflow of the commercially available dedicated post-processing software syngo.via version VB60A (Siemens Healthineers) was used for image analysis. All readers were blinded to the histopathological polyp class as well as patient identifier data such as age and gender. None of the readers were involved in the CT colonography dataset preparation. Before the actual reading, a training session was performed with an individual reading of 5 polyp image series. The polyp image series used for training were not included in the final study population. Colorectal polyps were presented in random order. Polyp segmentation masks were used for exact polyp localisation to avoid polyp detection bias, as this study focused on polyp classification and not polyp detection. If a polyp was visible in prone and supine positions, 2 polyp image series were evaluated per polyp. Polyps of all size categories and morphologies were evaluated.

The readers had to decide for each polyp image series whether the depicted polyp required endoscopic resection or could be monitored based on current guidelines recommending that colorectal polyps ≥ 6 mm should be resected (European Society of Gastrointestinal Endoscopy, European Society of Gastrointestinal and Abdominal Radiology United States Multi-Society Task Force on Colorectal Cancer, American Cancer Society, C-RADS) [14–17]. As it was shown that the interobserver agreement of radiologists using the Paris classification, which does not provide information on histopathology or prognosis of a finding at CT colonography, to categorise colorectal polyp morphology beyond size is only fair and consequently questionable to use at CT colonography, no further imaging criteria were mandatory [30]. The readers could measure polyp size on two-dimensional, multiplanar image reconstructions. Polyp size was defined as the largest polyp diameter, excluding the polyp stalk if visible, using a standard window width of 2000 HU and a window level of 2200 HU [23].

Colorectal polyp analysis comprised an unassisted and AI-assisted reading phase in a cross-sectional comparison study design [23, 31]. In the first phase, defined as unassisted reading, readers evaluated a polyp and assigned a respective recommendation (subsequent polypectomy: yes or no) without access to the AI model. Once the unassisted reading was complete, the result was locked and could not be changed anymore. In the second phase, defined as AI-assisted reading, readers re-evaluated the polyp with access to the finding of the AI model, which labelled the polyp as “adenomatous” or “non-adenomatous”, and confirmed or corrected their initial recommendation (subsequent polypectomy: yes or no) based on the prediction of the AI model. A correction of the initial recommendation based on the finding of the AI model was not mandatory.

### Histopathological reference standard

The recommendation “polypectomy: no” was considered correct if the corresponding histopathological report classified the depicted polyp as “regular mucosa” or “hyperplastic polyp”. The recommendation “polypectomy: yes” was considered correct if the corresponding histopathological report classified the depicted polyp as “tubular adenoma”, “tubulovillous adenoma”, “villous adenoma”, or “adenocarcinoma” [2, 5]. Only colorectal polyps unequivocally assignable to the corresponding histopathologic report were included in this study.

### Statistical analysis

**Fig. 2:**
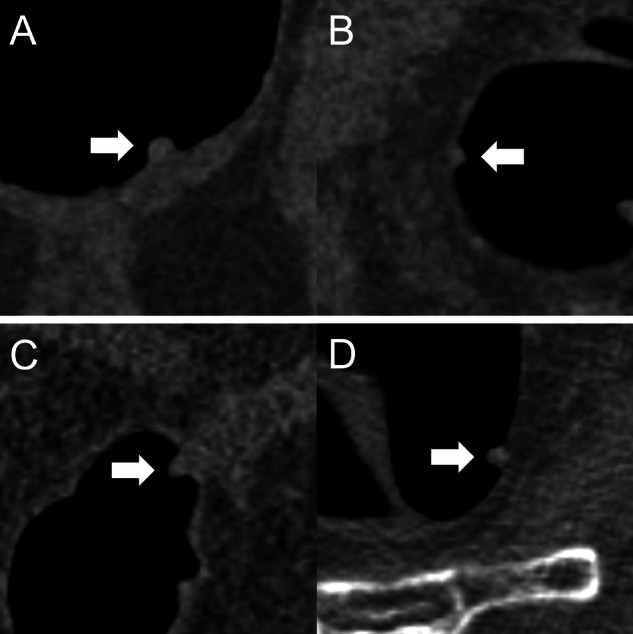
**A** A 9 mm tubular adenoma which was classified by all readers as “polypectomy: yes”. The initial recommendation was confirmed based on the prediction “adenomatous” of the AI model. **B** A 6 mm hyperplastic polyp which was classified by all readers as “polypectomy: no”. The initial recommendation was confirmed based on the prediction “non-adenomatous” of the AI model. **C** An 8 mm tubular adenoma which was classified by three readers as “polypectomy: no”. The initial recommendation was corrected by all three readers based on the prediction “adenomatous” of the AI model. **D** A 7 mm hyperplastic polyp which was classified by two readers as “polypectomy: yes”. The initial recommendation was corrected by both readers based on the prediction “non-adenomatous” of the AI model

With histopathology as the reference standard, a recommendation was considered correct if subsequent endoscopic resection of an adenomatous polyp was recommended or subsequent endoscopic resection of a non-adenomatous polyp was not recommended. A recommendation was considered incorrect if subsequent endoscopic resection of a non-adenomatous polyp was recommended, or subsequent endoscopic resection of an adenomatous polyp was not recommended (Fig. 2).

Diagnostic performance of unassisted, AI-assisted readings and AI-only readings was evaluated using accuracy, sensitivity, and specificity with a default classification threshold of 0.5. Inter-reader agreement between radiologists was quantified using Fleiss’ kappa. To account for individual effects from multiple readers generalised mixed effects models were used to test for differences in the unassisted and AI-assisted reading results.

## Results

### Study population

77 colorectal polyps in 59 patients comprising 118 polyp image series (56/118 (47%) supine position, 62/118 (53%) prone position) were evaluated unassisted and AI-assisted by 5 independent readers, resulting in a total of 1180 recommendations (subsequent polypectomy: yes or no). In 9/118 (8%) polyp images series the depicted polyps were 5 mm or smaller, in 56/118 (47%) between 6 and 9 mm, and in 53/118 (45%) 10 mm or larger, measuring the maximum three-dimensional diameter through the polyp [18]. In 18/118 (15%) polyp images series the depicted polyps were pedunculated, in 92/118 (78%) sessile, in 4/118 (3%) flat, and in 4/118 (3%) carcinomatous [18]. In 39/118 (33%) polyp image series the depicted polyps were classified as non-adenomatous (hyperplastic polyp or regular mucosa), of which 8/39 (21%) were 5 mm or smaller, 26/39 (67%) were between 6 and 9 mm, and 5/39 (13%) were 10 mm or larger [18]. In 79/118 (67%) polyp image series the depicted polyps were classified as adenomatous, of which 1/79 (1%) was 5 mm or smaller, 30/79 (38%) were between 6 and 9 mm, and 48/79 (61%) were 10 mm or larger [18].

### Statistical analysis

**Fig. 3:**
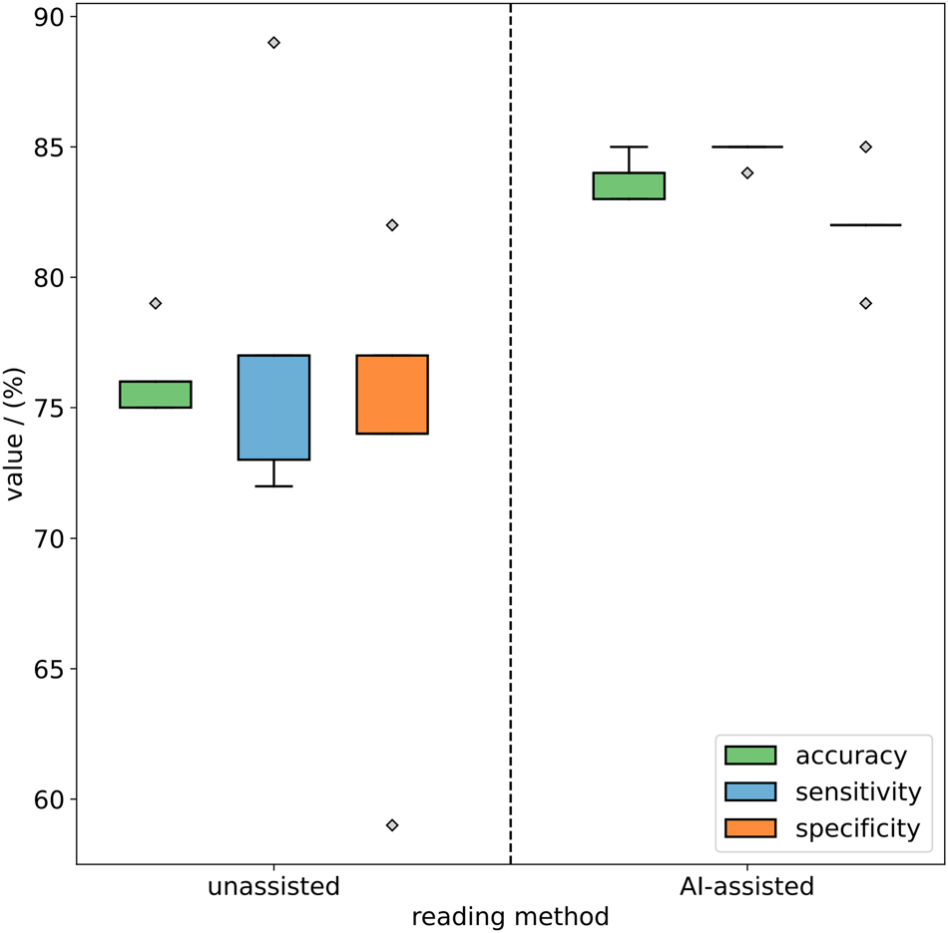
Accuracy (green), sensitivity (blue), and specificity (orange) distributions of unassisted (left) and AI-assisted (right) readings from all five readers for selecting colorectal polyps at CT colonography eligible for subsequent endoscopic resection

**Fig. 4:**
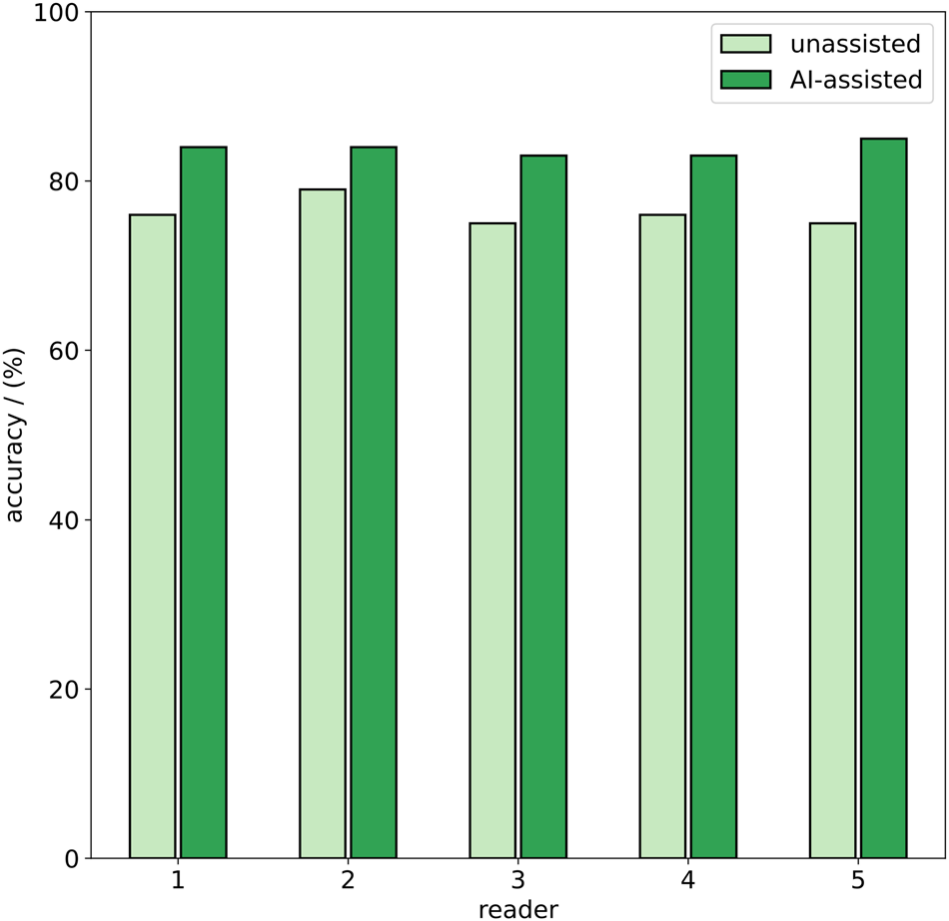
Comparison of reading accuracy in the selection of colorectal polyps on CT colonography that are eligible for subsequent endoscopic resection for unassisted (light green) and AI-assisted readings (dark green)

In unassisted readings, radiologists achieved an accuracy of 76% (standard deviation (SD) = 1%), a sensitivity of 78% (SD = 6%) and a specificity of 73% (SD = 8%) on average on selecting polyps eligible for polypectomy (Figs. 3 and 4). In AI-assisted readings, radiologists achieved an accuracy of 84% (SD = 1%), a sensitivity of 85% (SD = 1%) and a specificity of 82% (SD = 2%) in selecting polyps eligible for polypectomy. A detailed summary of reading performance by each radiologist is provided in Table 1. The random-forest machine-learning model alone achieved an accuracy of 83%, a sensitivity of 82%, and a specificity of 85% in selecting polyps eligible for polypectomy. The difference between unassisted and AI-assisted reading results was significant (*p* < 0.001). Inter-reader agreement between the 5 radiologists was Fleiss’ kappa 0.69 (0 = no agreement, 1 = full agreement) in unassisted readings and Fleiss’ kappa 0.92 in AI-assisted readings. The reading accuracy, sensitivity, and specificity for the three different size categories ≤ 5 mm, 6–9 mm, and ≥ 10 mm are presented in Table 2.

**Table 1:** Performance of each reader in unassisted reading and AI-assisted reading in selecting colorectal polyps at CT colonography eligible for subsequent endoscopic resection

Reader	Unassisted reading			AI-assisted reading		
	Accuracy	Sensitivity	Specificity	Accuracy	Sensitivity	Specificity
**1**	76%	77%	74%	84%	85%	82%
**2**	79%	89%	59%	84%	85%	82%
**3**	75%	72%	82%	83%	84%	82%
**4**	76%	77%	74%	83%	85%	79%
**5**	75%	73%	77%	85%	85%	85%

**Table 2:** Reading accuracy, sensitivity and specificity in different polyp size categories (≤ 5 mm, 6–9 mm, ≥ 10 mm)

	Unassisted reading			AI-assisted reading		
	≤ 5 mm	6–9 mm	≥ 10 mm	≤ 5 mm	6–9 mm	≥ 10 mm
Accuracy	100%	65%	88%	100%	77%	90%
Sensitivity	100%	56%	94%	100%	68%	97%
Specificity	100%	75%	20%	100%	89%	05%

A total number of 66 reading decisions were changed in the AI-assisted reading compared to the unassisted reading. Each reader changed 13.2 (SD = 1.9) decisions on average. In total, the changed decision was correct according to the histopathological reference standard in 55/66 (83%) cases and false in 11/66 (17%) cases. For polyps ≤ 5 mm no decision was changed. For polyps of 6–9 mm the changed decision was correct in 47/54 (87%) cases and false in 7/54 (13%) cases. For polyps ≥ 10 mm the changed decision was correct in 8/12 (67%) cases and false in 4/12 (33%) cases. A detailed summary of changed reading decisions made by each radiologist is provided in Table 3.

**Table 3:** Changed reading recommendations (subsequent polypectomy: yes or no) of each reader after having access to the findings of the AI model, which labelled colorectal polyp as “adenomatous” or “non-adenomatous”

Reader	Number of changes	Correct changes	False changes
**1**	13	11 (85%)	2 (15%)
**2**	14	10 (71%)	4 (29%)
**3**	13	11 (85%)	2 (15%)
**4**	10	9 (90%)	1 (10%)
**5**	16	14 (88%)	2 (12%)

## Discussion

In this study we evaluated the effect of AI-assisted differentiation of non-adenomatous from adenomatous colorectal polyps as a second reader at CT colonography. Unassisted and AI-assisted reading results were significantly different (*p* < 0.001). Compared with unassisted reading based on current guidelines, the AI-assisted reading had a higher accuracy (76% +/− 1% vs. 84% +/− 1%), sensitivity (78% +/− 6% vs. 85% +/− 1%), and specificity (73% +/− 8% vs. 82% +/− 2%) in selecting polyps eligible for polypectomy in correlation with the histopathological reference standard. Furthermore, inter-reader agreement between the five board-certified radiologists was improved in the AI-assisted reading (Fleiss’ kappa 0.69 vs. 0.92).

Previous studies indicate that AI-assisted CT colonography analysis enables the classification (non-adenomatous vs. adenomatous) of colorectal polyps [18, 19, 32]. For instance, Song et al reached an AUC of 0.85 in characterising colorectal polyps ≥ 8 mm at CT colonography using a Haralick texture analysis-based model [19]. Aman et al showed that the classification of colorectal polyps in CT colonography using content-based image retrieval achieved a significantly higher (*p* = 0.048) AUC of 0.76 as opposed to a polyp size–only approach with an AUC of 0.66 [32]. The radiomics-based random-forest model used in the present study enabled a robust differentiation of non-adenomatous and adenomatous colorectal polyps of all sizes at CT colonography with an AUC of 0.91 in an external test set [18]. However, an assessment of the effects of these AI models on therapy management in a clinical scenario is lacking. The present study adds to the field by evaluating the potential effects of AI-assisted polyp classification in CT colonography as a second reader. Our results indicate that it could further increase the clinical impact of CT colonography by enabling a more precise selection of patients who would benefit from subsequent endoscopic polypectomy.

CT colonography guidelines recommend the resection of colorectal polyps ≥ 10 mm and colorectal polyps of 6–9 mm depending on age or comorbidity, as endoscopic referral for polyps with a size of ≤ 5 mm at screening CT colonography has been shown to have poor cost-effectiveness with $464,407 per life-year gained, compared to $59,015 for polyps with a size of 6–9 mm, and $151 cost savings per person for polyps with a size of ≥ 10 mm [14–17, 33]. AI-assisted polyp classification as a second reader could potentially increase the effectiveness of endoscopic referral after CT colonography by aiding the radiologist to differentiate between adenomatous and non-adenomatous colorectal polyps.

This study had limitations. The readers were blinded to the information whether several polyp image series (i.e., prone and supine) belonged to one polyp or several polyps to one patient to ensure good comparability with the purely image-based AI model. However, correlations within multiple image series of one polyp or within multiple polyps of one patient cannot be ruled out. Including this information in AI-based polyp analysis should be evaluated in future studies. The AI model used in this study was based on manual polyp segmentation. Considering the rather small size of colorectal polyps, manual segmentation can be performed in a reasonable timeframe, but the further implementation of automated polyp segmentation into the AI workflow is required to ensure flawless implementation in clinical practice. For study purposes, four adenocarcinoma image series were included in this study. Yet a large lesion size implicates treatment regardless of the finding of the AI model. No serrated adenomas were included in this study as the prevalence of serrated adenomas is low. Based on the adenoma-carcinoma sequence the recommendation “polypectomy: yes” for tubular adenoma of all sizes was considered as correct in this study [2, 5]. However, an AI-assisted CT colonography approach focusing on advanced adenoma should be evaluated in future studies.

In conclusion, AI-based characterisation beyond the size and morphology of colorectal polyps at CT colonography as a second reader might enable a more precise selection of polyps eligible for subsequent endoscopic resection. This study indicates that integrating an AI tool for colorectal polyp classification in CT colonography could further improve radiologists’ therapy management. However, further studies are needed to confirm this finding and histopathologic polyp evaluation is still mandatory.

## Data Availability

Data generated or analysed during the study are available from the corresponding author by request.

## Abbreviations

C-RADS: CT Colonography Reporting and Data System
TCIA: The Cancer Imaging Archive
